# Hyperactive Behavior and Altered Brain Morphology in Adult Complement C3a Receptor Deficient Mice

**DOI:** 10.3389/fimmu.2021.604812

**Published:** 2021-02-22

**Authors:** Andrea Pozo-Rodrigálvarez, Roosa Ollaranta, Jenny Skoog, Milos Pekny, Marcela Pekna

**Affiliations:** ^1^Laboratory of Regenerative Neuroimmunology, Department of Clinical Neuroscience, Institute of Neuroscience and Physiology, Sahlgrenska Academy at the University of Gothenburg, Gothenburg, Sweden; ^2^Laboratory of Astrocyte Biology and CNS Regeneration, Department of Clinical Neuroscience, Institute of Neuroscience and Physiology, Sahlgrenska Academy at the University of Gothenburg, Gothenburg, Sweden; ^3^Florey Institute of Neuroscience and Mental Health, Parkville, VIC, Australia; ^4^University of Newcastle, Newcastle, NSW, Australia

**Keywords:** autism spectrum disorder, attention deficit hyperactivity disorder, C3aR, the complement system, neurodevelopment

## Abstract

The C3a receptor (C3aR) is a seven trans-membrane domain G-protein coupled receptor with a range of immune modulatory functions. C3aR is activated by the third complement component (C3) activation derived peptide C3a and a neuropeptide TLQP-21. In the central nervous system (CNS), C3aR is expressed by neural progenitors, neurons as well as glial cells. The non-immune functions of C3aR in the adult CNS include regulation of basal neurogenesis, injury-induced neural plasticity, and modulation of glial cell activation. In the developing brain, C3aR and C3 have been shown to play a role in neural progenitor cell proliferation and neuronal migration with potential implications for autism spectrum disorder, and adult C3aR deficient (*C3aR*^−/−^) mice were reported to exhibit subtle deficit in recall memory. Here, we subjected 3 months old male *C3aR*^−/−^ mice to a battery of behavioral tests and examined their brain morphology. We found that the *C3aR*^−/−^ mice exhibit a short-term memory deficit and increased locomotor activity, but do not show any signs of autistic behavior as assessed by self-grooming behavior. We also found regional differences between the *C3aR*^−/−^ and wild-type (WT) mice in the morphology of motor and somatosensory cortex, as well as amygdala and hippocampus. In summary, constitutive absence of C3aR signaling in mice leads to neurodevelopmental abnormalities that persist into adulthood and are associated with locomotive hyperactivity and altered cognitive functions.

## Introduction

The complement system is a powerful effector of the innate immune system. C3aR is a seven trans-membrane domain G-protein coupled receptor expressed on myeloid cells as well as several types of non-myeloid cell types (1) and exerts a range of both pro-inflammatory and anti-inflammatory functions (2). C3aR is activated by C3a, a 9 kDa fragment generated by the proteolytic cleavage of the third complement component (C3) (3), and a vascular growth factor-derived neuropeptide TLQP-21 (4). In the brain, C3aR is expressed by neural progenitor cell (5, 6), neurons (7–10) as well as glial cells (7, 8, 11–13).

The non-immune functions of C3aR in the adult CNS include regulation of basal and ischemia-induced neurogenesis (5, 14), synaptic strength and dendritic complexity (15), neuroprotection (16), injury-induced neural plasticity (17), and modulation of glial cell activation (13, 18); reviewed in (19). *In vitro*, C3a modulates migration of neural progenitor cells and stimulates their neuronal maturation (20). In the developing brain, C3aR plays a role in neural progenitor cell proliferation (6), and C3aR and C3 cleavage products are required for normal neuronal migration (21), which is an essential process for normal brain formation and establishment of neural circuits. Disturbed complement activity disrupts migration of pyramidal neurons from the ventricular zone to their correct laminar position in the cortical plate, leading to disordered layering of the developing cortex. This developmental defect can be rescued by pharmacological activation of C3aR (21). Deficiency of C3 results in reduced activity of small GTPase Rac1, with consequent cell cycle defects and premature neuronal differentiation (22). Given that C3aR activation by C3a was shown to regulate Rac1 activity in migrating crest cells (23), the regulation of Rac1 activity by C3 in migrating neurons is conceivably mediated through the C3a-C3aR signaling. The neurodevelopmental functions of C3aR signaling may thus be relevant for the understanding of cellular and molecular mechanisms underlying intellectual disabilities, schizophrenia, and autism (21). Indeed, adult *C3aR*^−/−^ mice were reported to exhibit subtle deficit in recall memory (6), but a detailed analysis of their behavior and brain morphology has been lacking.

The objective of the present study was to perform a more detailed investigation of the brain morphology of adult *C3aR*^−/−^ mice, determine whether the previously reported disordered layering of the developing cortex due to disturbed C3aR signaling persists into the adulthood, and examine the behavior of *C3aR*^−/−^ mice with focus on locomotor activity, motor function, memory, and autism-like repetitive behavior.

## Materials and Methods

### Animals

*C3aR*^−/−^mice (24) were backcrossed onto the C57BL/6J genetic background (Jackson Laboratories) for 10 generations. Heterozygous mice were then intercrossed to generate homozygous *C3aR*^−/−^ mice. WT C57BL/6J mice served as controls. Male, 2-month-old mice were used. Mice were housed at Experimental Biomedicine (EBM), Sahlgrenska Academy, University of Gothenburg with a 12 h light/dark cycle and free access of food and water. All animal experiments were conducted according to protocols approved by the Gothenburg Ethics Committee.

### Open Field

Mice were individually placed in the square arena, and allowed to explore the apparatus for 10 min while they were recorded to study their locomotor and exploratory activities (25). The arena was divided in 16 equal areas, considering the four central squares (25% of the total arena) as the central zone and the surrounding areas as the peripheral zone. The parameters measured during the duration of the stay were speed (cm/s), activity, defined as the fraction of time in which the speed exceeded 0.2 cm/s, total distance covered in each zone (cm), the number of rearings, and the number of grooming episodes. The data were analyzed with the Viewer^3^ video tracking system (Biobserve, Bonn, Germany).

### Object Recognition

The object recognition test is based on the innate preference of mice to explore a novel object rather than a familiar one. The test was performed as previously described (25). Mice were habituated to the room 3 days before the test. During the familiarization session, two identical objects were used. Six hours later one of the objects was replaced by a different one for the short-term memory (STM) assessment. Twenty four hours later, long-term memory (LTM) was assessed by replacing the same object with a novel one. During each session, the mice were free to explore the objects for 10 min and then returned to home cage. Between mice, the arena and objects were cleaned with 70% ethanol. Exploration was defined as directing the nose to the object at a <2 cm distance or touching the object with the nose or forepaws. Mice that showed unequal exploration of the two identical objects or failed to explore each object for at least 20 s during the familiarization session were excluded. Viewer^3^ video tracking system was used for data analysis.

### Contextual and Cued Trace Fear Conditioning Test

Contextual and cued trace fear conditioning test was used to test the amygdala- and hippocampus-dependent associative cognition as described previously (25). Briefly, mice were acclimatized to the room for 1 h and habituated for 10 min to the conditioning chamber Automatic Reflex Conditioner (Ugo Basile, Gemonio, Italy). In the training session 24 h later, individual mice were subjected to the conditioning cue (a 70 dB, 670 Hz tone) for 20 s, followed by a trace period of 18 s and a foot shock (0.5 mA, 2 s). This sequence was delivered 8 times at 60 s intervals. Thereafter, mice returned to home cage. Between mice the chamber was cleaned with 70% ethanol. The next day, contextual response was assessed in the same chamber (old context) during 3 min. One to three hours later, the mice were placed in a new context (a plastic cylinder with striped walls and plastic floor, and novel odor of vanilla extract). After 3 min of exploration (pre-CS), the auditory cue was presented 4 times for 20 s at 60 s intervals (CS). During each session, mice were recorded and freezing behavior, defined as the absence of all body movements other than those associated with respiration, was scored manually every 5th second. Results were expressed as the relative fraction of total time spent in freezing (%).

### Brain Collection and Processing

The mice were deeply anesthetized with thiopental [Pentothal Sodium (0.01 ml/g body weight), Hospira, Illinois, USA] and transcardially perfused with PBS, followed by 4% paraformaldehyde (PFA) in 0.1 M PBS. Brains were removed and post-fixed in 4% PFA at 4°C for 24 h followed by 70% ethanol for 24 h. Tissue was processed using an automatic tissue processor (SAKURA Tissue TeK VIP 3000, Tournai, Belgium) and embedded in paraffin. Brains were cut into 8-μm serial coronal sections using a sliding microtome (Microm HM 450, Thermo Scientific, Massachusetts, USA), attached to silane coated slides and dried at room temperature (RT).

### Immunohistochemistry

Neuronal nuclei (NeuN), cut like homeobox 1 (Cux-1), and T-box brain transcription factor 1 (Tbr-1) were visualized in rostral (between Bregma 1.70 and 1.34 mm) and caudal (between Bregma −0.22 and −0.82 mm) motor cortex, primary and secondary somatosensory cortex by immunohistochemistry. Briefly, sections were deparaffinized and rehydrated followed by heat-induced antigen retrieval with 0.01 M citrate buffer (pH 6, 0.05% Tween 20) for NeuN and Cux-1, or 0.01 M Tris- 1 mM EDTA (pH 9, 0.05% Tween 20) buffer for Tbr-1. After washing with PBS-T (0.05% Tween 20), non-specific protein binding was reduced by incubation with blocking buffer (4% normal donkey serum in PBS-T for 1 h at room temperature (RT). Tissue was then incubated overnight at 4°C with primary antibody [anti-NeuN biotinylated (1:200, MAB 377B, Millipore, MA, USA), anti-Cux-1 (1:1,000, ab54583, Abcam), anti-Tbr-1 (1:1,000, ab31940, Abcam)], in blocking buffer. One section per slide was incubated only with blocking buffer without primary antibody and used as a negative control. Next day, the sections were washed with PBS-T and incubated with secondary antibody [rabbit-anti mouse biotinylated Ig (1:200, E0354, DAKO, Stockholm, Sweden) for Cux-1, donkey-anti rabbit biotinylated Ig (1:200, 711-065-152, Jackson ImmunoResearch Inc., PA, USA) for Tbr-1], in blocking buffer for 1 h at RT. After washing, Neun immuonstained sections were incubated with Streptavidin-Cy3 (1:100, S6402, Sigma-Aldrich, Missouri, USA) in blocking buffer for 1 h at RT, washed, mounted with ProLong Gold (P36931, Life Technologies, CA, USA), coverslipped and dried for 24 h before being sealed with nail polish. For Cux-1 and Tbr-1 immunostaining, sections were incubated with avidin/biotin complex (VECTASTAIN® Elite ABC kit, PK-6100, Vector Laboratories Inc., CA, USA) followed by diaminobenzidine (DAB) Substrate Kit (SK-4100, Vector Laboratories Inc., CA, USA) according to manufacturer's instructions. After washing, sections were dehydrated (70% EtOH 2 min, 95% EtOH 2 min, 100% EtOH 2 min), and cleared with xylene for 5 min. Slides were mounted with Vecta Mount medium (H-5000, Vector Laboratories Inc., CA, USA) and coverslipped.

Cells were counted using ImageJ 1.46r software. NeuN positive cells were counted on immunofluorescence images obtained with a 10x objective. Cux-1 and Tbr-1 positive cells were counted on bright field images obtained with a 20x objective (Nikon Eclipse 80i). Three sections (200 μm apart) per rostral and caudal cortex, respectively, and animal were used for the analysis and the data were presented as density (cells/μm^2^). Cortical thickness was measured on NeuN stained images obtained with a 4x objective.

### Morphometric Analysis of Amygdala, Hippocampus, and Brain Volume

For the morphometric analysis, sections were stained with hematoxylin and eosin. Wide-field microscope with a 4x objective (Nikon Eclipse 80i, Nikon Instruments Inc., Tokyo, Japan) was used to obtain images of 3 brain sections per mouse and level at three levels −1.22, −1.70, and −2.18 mm relative to Bregma. ImageJ 1.46r software was used to trace around the hippocampus, basolateral amygdaloid nucleus, basomedial amygdaloid nucleus, medial amygdaloid nucleus post erodorsal, and the hemispheres. Volumes were calculated according to the Cavalieri's principle, where V = *Σ*A × P × T (26).

### Statistical Analysis

Statistical analyses were done with Microsoft Excel and GraphPad Prism 7.0 (GraphPad Software Inc., San Diego, CA, USA). Two-tailed unpaired Student's *t*-test was used for comparisons between two groups. Two-way Analysis of Variance (ANOVA) followed by a Tukey's multiple comparisons *post-hoc* test was used to analyze time and distance in the open field, time and maximum speed in the rotarod test, relative exploration time and relative visitation of the objects in the object recognition test, as well as freezing time during training and in the new context in the fear conditioning test. Differences were considered significant at *p* < 0.05. Data are presented as mean ± standard error of the mean (SEM).

## Results

### *C3aR^−/−^* Mice Are Hyperactive and Exhibit Signs of Increased Anxiety

**Figure 1:**
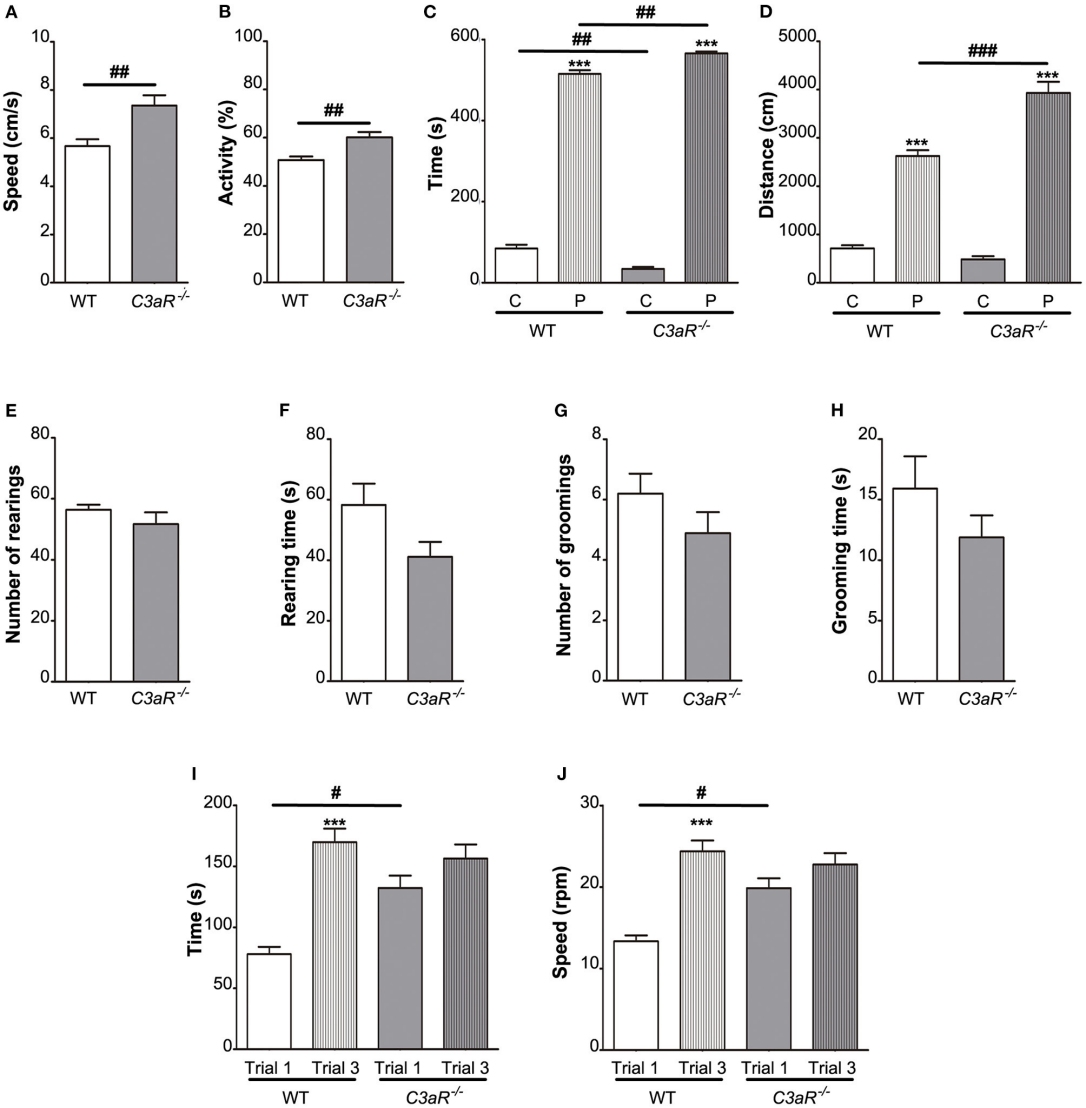
*C3aR*^−/−^ mice are hyperactive with signs of increased anxiety. Open field assessment of locomotion, anxiety-like and repetitive behavior. **(A)** speed, **(B)** relative activity presented as % of the total time, **(C)** time in the center, C, and periphery, P, of the arena, **(D)** distance covered, **(E)** number of rearings, **(F)** rearing time, **(G)** number of grooming episodes, **(H)** grooming time; *n* = 19 (WT) and 9 (*C3aR*^−/−^). Rotarod assessment of motor function, coordination, and balance **(I)** time, **(J)** max speed; *n* = 9 (WT) and 11 (*C3aR*^−/−^). Mean ± SEM. ^***^p < 0.001 for within group comparisons; ^#^*p* < 0.05, ^##^*p* < 0.01, and ^###^*p* < 0.001 for between group comparisons. Two-tailed unpaired Student's *t*-test **(A,B,E–H)**, and two-way ANOVA followed by a Tukey's multiple comparisons *post-hoc* test **(C,D,I,J)**.

To assess the overall locomotor activity, we subjected the *C3aR*^−/−^ and WT mice to the open field test. We found that the *C3aR*^−/−^ mice moved at higher speed (*p* < 0.01; Figure 1A), and spent more time in motion (*p* < 0.01; Figure 1B). Both groups preferred the peripheral area of the open field box over the central area, however, the distance covered in the centrum was shorter in the *C3aR*^−/−^ group (Figure 1C). In contrast, in the periphery the *C3aR*^−/−^ mice moved more than the WT mice (*p* < 0.01 and *p* < 0.001 for time and distance covered, respectively; Figures 1C,D). We did not observe any difference between the two groups in rearing or self-grooming activities (Figures 1E–H). Next, we assessed the motor function, coordination, and balance using the rotarod. The *C3aR*^−/−^ mice performed better on the test during the first trial (*p* < 0.05 for both time and speed), but in contrast to the WT mice, their performance did not improve between trial 1 and 3 (Figures 1I,J). Jointly, these results show that the *C3aR*^−/−^ mice are hyperactive with signs of increased anxiety, but they do not exhibit autism-like repetitive behavior.

### *C3aR^−/−^* Mice Have Impaired Short-Term Memory

**Figure 2:**
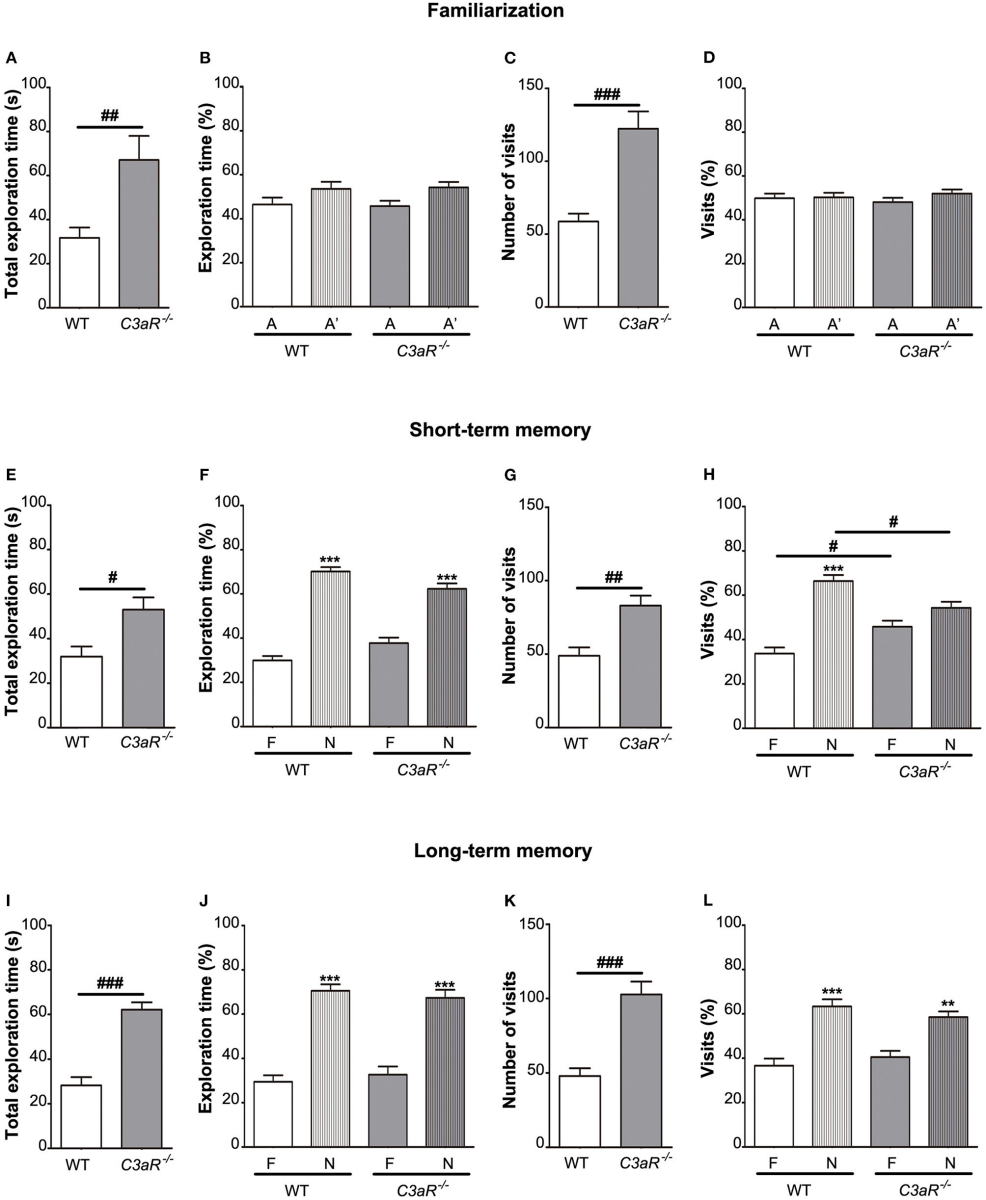
*C3aR*^−/−^ mice have impaired short-term memory. Object recognition test assessment of short and long-term memory. **(A)** time spent exploring individual objects, **(B)** relative exploration time, **(C)** number of visits, and **(D)** relative visitation of the objects during familiarization, short-term memory testing 6 h later **(E–H)** and long-term-memory testing 24 h later **(I–L)**. A, A′, identical objects used during the familiarization phase; F, familiar object; N, novel object used during the testing phases. *n* = 11 (WT) and 8 (*C3aR*^−/−^). Mean ± SEM. ^**^p < 0.01 and ^***^p < 0.001 for within group comparisons; ^#^*p* < 0.05, ^##^*p* < 0.01, and ^###^*p* < 0.001 for between group comparisons. Two-tailed unpaired Student's *t*-test **(A,C,E,G,I,K)** and two-way ANOVA followed by a Tukey's multiple comparisons *post-hoc* test **(B,D,F,H,J,L)**.

Next, we used object recognition test to assess short and long-term memory. In both familiarization and test sessions, the *C3aR*^−/−^ mice exhibited increased explorative behavior, as shown by total exploration time (*p* < 0.01, *p* < 0.05, and *p* < 0.001 for familiarization, short and long term memory test, respectively) as well as total number of visits to the objects (*p* < 0.001, *p* < 0.01, and *p* < 0.001 for familiarization, short and long term memory, respectively; Figures 2A–K). During the familiarization session, the mice in both groups explored the two objects to an equal extent (Figures 2B,D). Mice in both groups spent more time exploring the novel object in the short as well as long-term memory test (*p* < 0.001; Figures 2F,J), however, in the short-term memory test, in contrast to the WT group, the *C3aR*^−/−^ mice did not discriminate between the familiar and novel object in terms of the relative number of visits (Figure 2H). In the long-term memory test, there was no difference between the groups in the relative exploration of the novel vs. familiar object (Figures 2J,L). These results confirm the hyperactive behavior of the *C3aR*^−/−^ mice as observed in the open field test and point to the impairment in their short-term memory.

**Figure 3:**
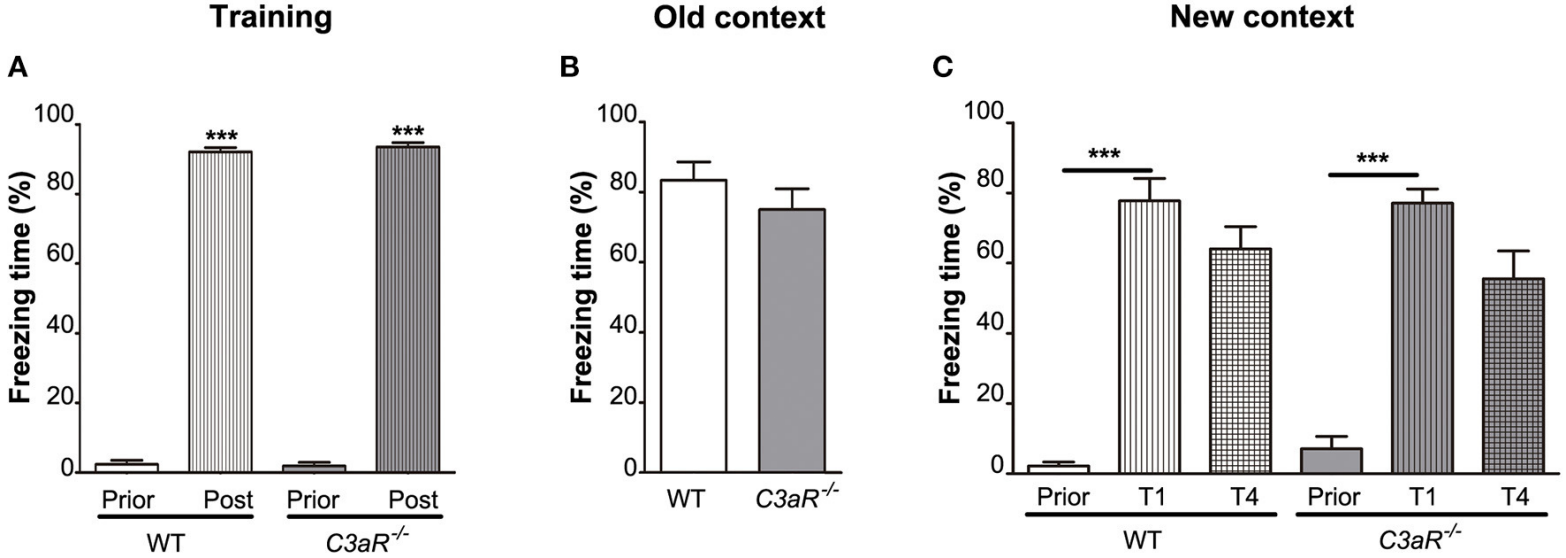
*C3aR*^−/−^ mice exhibit normal formation and extinction of fear memory. Assessment of learning and memory by contextual/cued fear conditioning test. Relative time spent in freezing behavior during **(A)** training, and testing in **(B)** the old context without auditory cue, **(C)** new context in response to auditory cue. Prior, post, before and after electric shock, respectively **(A)**; Prior, T1, T4, before and after the first and fourth exposure to auditory cue, respectively **(C)**. *n* = 19 (WT) and 9 (*C3aR*^−/−^). Mean ± SEM. ^***^p < 0.001 for within group comparisons. Two-tailed unpaired Student's *t*-test **(B)** and two-way ANOVA followed by a Tukey's multiple comparisons *post-hoc* test **(A,C)**.

Using fear conditioning test, we did not find any difference between the *C3aR*^−/−^ and WT mice in their freezing behavior during the training (Figure 3A) and when placed in the old context without the auditory cue (Figure 3B). None of the groups showed a reduction in freezing response between the first and the fourth trial of exposure to the auditory cue in the new context (Figure 3C). These results suggest that memory formation and extinction as assessed by fear conditioning are not altered in the *C3aR*^−/−^ mice.

### *C3aR^−/−^* Mice Exhibit Regional Alterations in Cortical Morphology

**Figure 4:**
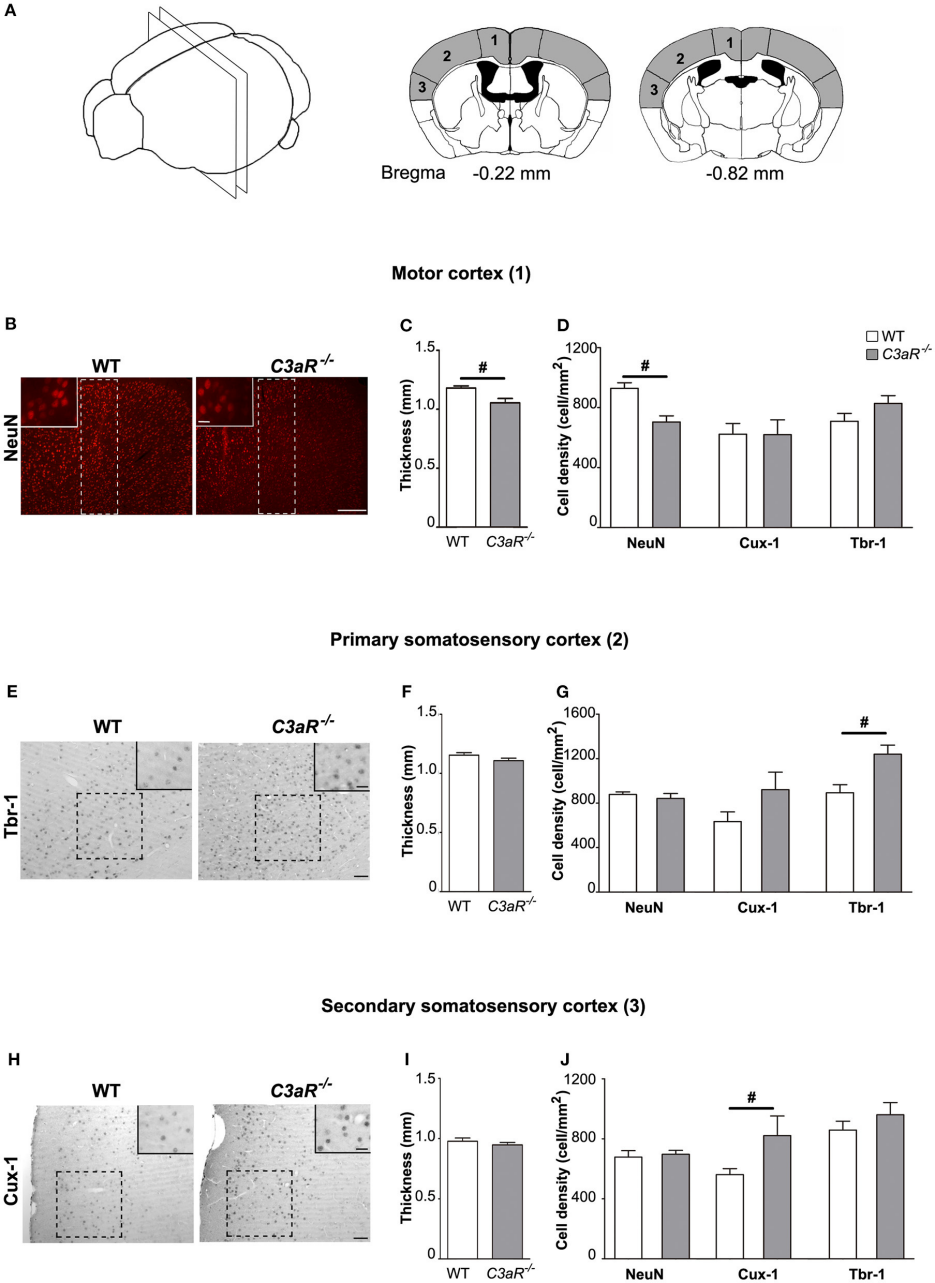
*C3aR*^−/−^ mice exhibit regional alterations in cortical morphology. Morphological assessment of the thickness of the positive cell layer and the density of NeuN positive, Cux-1 positive, and Tbr1 positive cells in the caudal motor and somatosensory cortex. **(A)** Schematic illustration of the cortical regions assessed including the distance from bregma. 1, motor cortex; 2, primary somatosensory cortex; 3, secondary somatosensory cortex. **(B)** Representative images of neurons visualized by antibody against NeuN, **(C)** thickness, and **(D)** neuronal cell density in the motor cortex. **(E)** Representative images of neurons visualized by antibody against Tbr-1, **(F)** thickness, and **(G)** neuronal cell density in the primary somatosensory cortex. **(H)** Representative images of neurons visualized by antibody against Cux-1, **(I)** thickness, and **(J)** neuronal cell density in the primary somatosensory cortex. Broken line rectangle in **(B)** and squares in **(E,H)** indicate the region of interest used for quantification. Insets show representative images taken with 40x objective. *n* = 12, 10, 11 (WT) and 8, 6, 7 (*C3aR*^−/−^) for NeuN, Tbr-1, and Cux-1 positive cells, respectively. Mean ± SEM. ^#^*p* < 0.05, Two-tailed unpaired Student's *t*-test. Scale bars **(B)** 100 μm; **(E,H)** 50 μm; insets, 20 μm.

Next, we used antibodies against NeuN, a pan-neuronal marker, ortholog of the Drosophila homeobox Cut gene, Cux-1, a transcription factor expressed predominantly in cortical neurons in layer II-IV (27), and transcription factor T-box, brain, 1 (Tbr-1), a marker of glutamatergic neurons in cortical layer VI (28), to visualize these neuronal populations on brain sections of *C3aR*^−/−^ and WT mice, measure the cortical thickness, and count neurons in the respective layers of the rostral and caudal motor and somatosensory cortex (Figure 4A). We did not find any difference between the groups in the thickness of the Tbr-1 and Cux-1 cortical layers at any level (data not shown). No differences between the groups were observed in the rostral cortex (data not shown). However, the thickness of caudal motor cortex and the neuronal cell density of caudal motor cortex were reduced in the *C3aR*^−/−^ mice (*p* < 0.05; Figures 4B–D). The *C3aR*^−/−^ mice showed also higher density of Tbr-1 neurons in the caudal primary somatosensory cortex (*p* < 0.05; Figures 4E,G), and higher density of Cux-1 neurons in the caudal secondary somatosensory cortex (*p* < 0.05; Figures 4H,J). The thickness of the somatosensory cortex did not differ between the groups (Figures 4F,I).

### *C3aR^−/−^* Mice Have Larger Amygdala and Hippocampus but Normal Brain Volume

**Figure 5:**
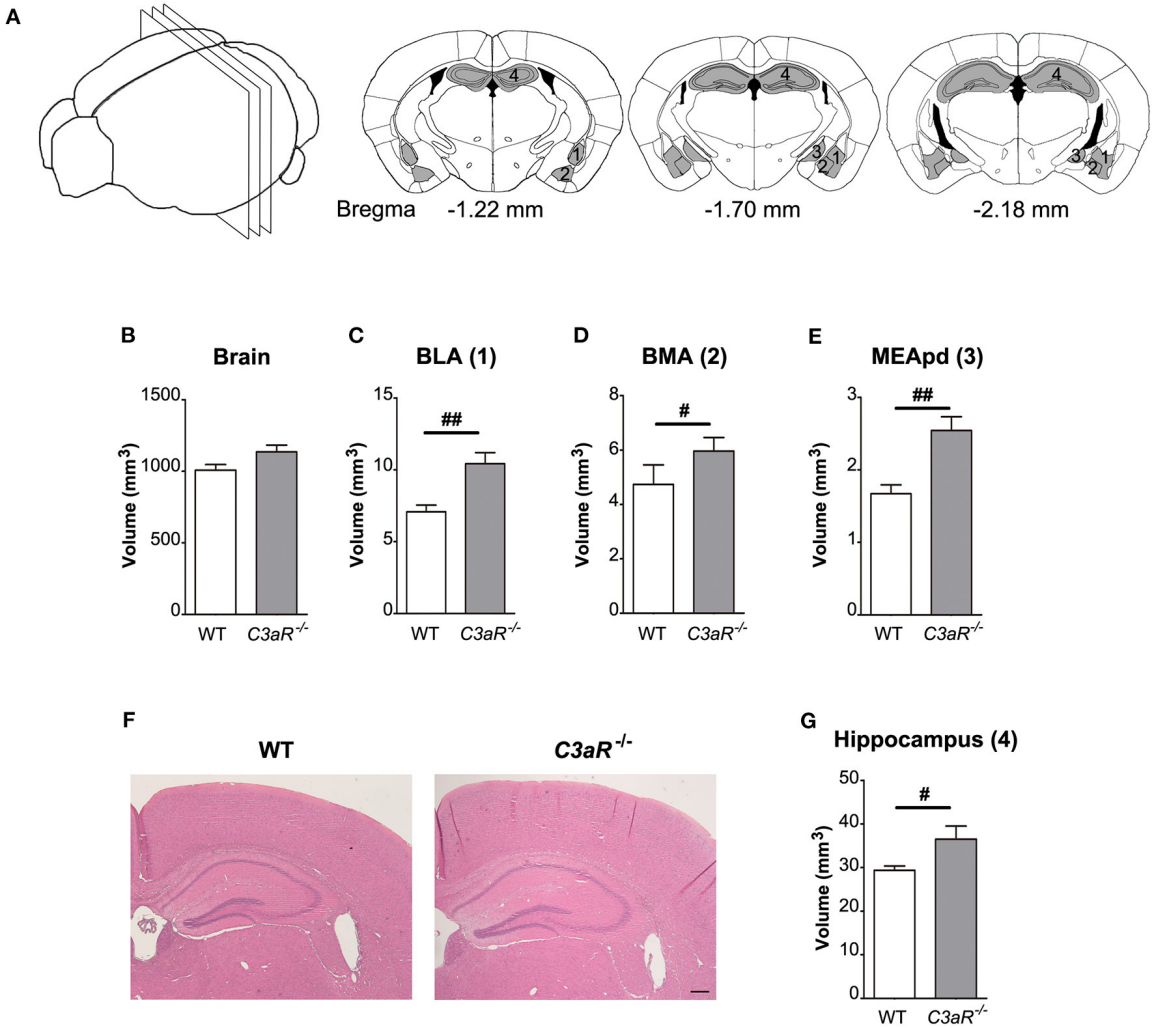
*C3aR*^−/−^ mice have larger amygdala and hippocampus but normal brain volume. Morphological assessment of the volume of the amygdala, the hippocampus, and the brain. **(A)** Schematic representation of the regions assessed and brain sections used for the quantification. Volume of **(B)** the whole brain between bregma −1.22 mm and −2.18 mm. **(C)** BLA, basolateral amygdalar nucleus; **(D)** BMA, basomedial amygdalar nucleus; **(E)** MEApd, medial amygdalar nucleus post-erodorsal; **(G)** hippocampus. **(F)** Representative images of hippocampus at bregma −1.70 mm; *n* = 11 per group; mean ± SEM. ^#^*p* < 0.05 and ^##^*p* < 0.01. Two-tailed unpaired Student's *t*-test. 1–4 in **(A)** indicate the location of the respective regions in **(B–E)**. Scale bar, 200 μm.

Next, we used hematoxylin-erythrosin stained sections to measure the volume of amygdala and hippocampus (Figure 5A), brain regions involved in the regulation of fear response, and learning and memory, respectively. While the total brain volume did not differ between the groups (Figure 5B), the *C3aR*^−/−^ mice had larger amygdala (*p* < 0.01 for basolateral amygdaloid nucleus, *p* < 0.05 for basomedial amygdaloid nucleus, *p* < 0.01 for medial amygdaloid nucleus post erodorsal) as well as hippocampus (*p* < 0.05), Figures 5C–G.

## Discussion

C3aR has emerged as a novel factor in the regulation of CNS development, including embryonic neural stem cell proliferation (6) and neuronal migration (20, 21), however, the adult *C3aR*^−/−^ mice were reported to be developmentally grossly normal with only a subtle defect in recall memory (6). Here we show that adult *C3aR*^−/−^ mice exhibit abnormal behavior and regional differences in brain morphology. These results point to non-redundant roles of C3aR signaling in brain development and function, roles that are not compensated by other factors.

First, we found that the *C3aR*^−/−^ mice are hyperactive as assessed by their locomotor activity in both the open field and object recognition test. In addition, the fact the *C3aR*^−/−^ mice spend more time than the WT mice in the periphery could indicate increased anxiety-like behavior in the former. These behavioral abnormalities are reminiscent of the main symptoms of attention deficit hyperactivity disorder (ADHD), a neurodevelopmental disorder of complex etiology that is characterized by hyperactivity, impulsiveness, and impaired attention that can persist into adulthood, with anxiety as a common comorbidity (29). Remarkably, the *C3aR*^−/−^ mice had larger amygdala, including basolateral amygdala, a brain region that has been implicated in the development of anxiety in rodents (30). Hyperactivity and cognitive impairment are also among the behavioral features of several animal models of schizophrenia (31). While there is a mounting body of evidence supporting the role of neuroinflammation in the pathophysiology of ADHD (32) and the role of aberrant complement activity in excessive synapse elimination and the development of schizophrenia (33), our findings of larger amygdala and abnormal behavior in *C3aR*^−/−^ mice point to the importance of basal complement activity and signaling through C3aR for normal brain development and function. The relevance of *C3aR*^−/−^ mice as a model of ADHD or schizophrenia merits further investigation.

Our results of impaired short-term memory in the *C3aR*^−/−^ mice are in line with the report by Coulthard et al. (6). Notably, while their brain volume was comparable to that of WT mice, the *C3aR*^−/−^ mice had larger amygdala and hippocampus, two brain regions involved in the cognitive processes assessed in our study. Given that C3aR signaling has been shown to play a role in multiple processes regulating neurodevelopment (6, 21) as well normal neuronal function of the adult brain (5, 15), the impaired cognitive performance in mice constitutively lacking C3aR may be caused by defects in neurogenesis, neuronal migration as well as synaptic function. In addition, C3aR is expressed also on astrocytes and microglia (7, 8, 11–13) and therefore dysfunction of glial cells, their cross-talk and communication with neurons are additional conceivable factors to consider as a potential mechanism linking C3aR signaling and cognitive function.

Our results also show that despite altered morphology of amygdala, one of the brain regions involved in the regulation of fear acquisition and extinction (30), memory formation, and extinction as assessed by fear conditioning were not altered in the *C3aR*^−/−^ mice. Given the proposed potential overlap between fear and anxiety circuits (30), our findings of selective effect of C3aR deficiency on anxiety-like behavior but not fear responses point to a distinct role of C3aR signaling in the neural networks controlling the generation of fear and anxiety states.

Aberrant neuronal migration has been put forward as part of the pathophysiology of autism-spectrum disorder, a neurodevelopmental condition with brain heterotopia, disrupted neuronal minicolumns, and changes in neuronal density and volume as the main neuroanatomical and histological features (34, 35). Stereotyped or repetitive behaviors are among the characteristic symptoms of autism, and self-grooming is used as a measure of repetitive behavior in rodents (36). Impaired neuronal migration due to dysfunction of the complement system has been proposed as one of the mechanisms leading to autism-spectrum disorder (21, 37). Recent findings of social interaction deficits and repetitive behavior induced by C3 knockdown in the prefrontal cortex of adult mice provide support for to a role of C3 deficiency in the pathophysiology of autism-spectrum disorder (38). Here we report that the adult *C3aR*^−/−^ mice exhibit subtle regional alterations in cortical organization, such as reduced thickness and neuronal density in the caudal motor cortex, and higher density of Tbr-1 and Cux-1 positive neurons in the caudal primary, and secondary somatosensory cortex, respectively. These abnormalities are consistent with the previously reported modulatory effects of C3a on migration of neural progenitor cells (20), the cortical migration defect caused by knockout and knockdown of C3 during embryonic development, and the rescue of this phenotype by C3aR agonist (21). Notably, ours is the first study that examined repetitive behavior in mice constitutively lacking C3aR. While the above findings of cortical organization abnormalities confirm the function of C3aR in the development of neocortex, our observation of self-grooming behavior of the *C3aR*^−/−^ mice that did not differ from that of WT mice, suggests that the disturbance of cortical layering in adult *C3aR*^−/−^ mice is too subtle to translate into apparent autistic behavior.

In summary, we show that constitutive absence of C3aR signaling in mice leads to abnormalities in the organization and morphology of the adult neocortex, amygdala, and hippocampus. The fact that these abnormalities are associated with locomotive hyperactivity and altered cognitive functions of the *C3aR*^−/−^ mice may have implications for the understanding of the cellular and molecular mechanisms underlying neurodevelopmental disorders such as ADHD and autism spectrum disorder.

## Data Availability Statement

The original contributions presented in the study are publicly available. This data can be found here: https://gin.g-node.org/10.12751/g-node.k8lx52.

## Ethics Statement

The animal study was reviewed and approved by Gothenburg Ethics Committee.

## Author Contributions

AP, MPekny, and MPekna conceptualized and designed the study. AP, RO, and JS performed the experiments. AP, MPekny, and MPekna wrote the manuscript. All authors read and approved the final version of the manuscript.

## Conflict of Interest

The authors declare that the research was conducted in the absence of any commercial or financial relationships that could be construed as a potential conflict of interest.
